# Finding the right mix: a framework for selecting seeding rates for cover crop mixtures

**DOI:** 10.1002/eap.2484

**Published:** 2021-11-24

**Authors:** K. Ann Bybee‐Finley, Stéphane Cordeau, Séverin Yvoz, Steven B. Mirsky, Matthew R. Ryan

**Affiliations:** ^1^ Sustainable Agricultural Systems Laboratory USDA‐ARS Beltsville Maryland 20705 USA; ^2^ Department of Plant, Soil and Microbial Sciences Michigan State University East Lansing Michigan 48915 USA; ^3^ Agroécologie AgroSup Dijon INRAE Univ. Bourgogne Univ. Bourgogne Franche‐Comté Dijon F‐21000 France; ^4^ Sustainable Cropping Systems Laboratory Section of Soil and Crop Sciences Cornell University Ithaca New York 14853 USA

**Keywords:** competition, cover crops, mixtures, multifunctionality, optimization, Pareto front, response surface design, yield–density model

## Abstract

Cover crop mixtures have the potential to provide more ecosystem services than cover crop monocultures. However, seeding rates that are typically recommended (i.e. seeding rate of monoculture divided by the number of species in the mixture) are non‐optimized and often result in the competitive species dominating the mixture, and therefore limiting the amount of ecosystem services that are provided. We created an analytical framework for selecting seeding rates for cover crop mixtures that maximize multifunctionality while minimizing seed costs. The framework was developed using data from a field experiment, which included six response surface designs of two‐species mixtures, as well as a factorial replacement design of three‐species and four‐species mixtures. We quantified intraspecific and interspecific competition among two grasses and two legume cover crop species with grass and legume representing two functional groups: pearl millet [*Pennisetum glaucum* (L.) R.Br.], sorghum sudangrass [*Sorghum bicolor* (L.) Moench × *Sorghum sudanense* (Piper) Stapf], sunn hemp (*Crotalaria juncea* L.), and cowpea [*Vigna unguiculata* (L.) Walp]. Yield–density models were fit to estimate intraspecific and interspecific competition coefficients for each species in biculture. The hierarchy from most to least competitive was sorghum sudangrass > sunn hemp > pearl millet > cowpea. Intraspecific competition of a less competitive species was the greatest when the biculture was composed of two species in the same functional group. Competition coefficients were used to build models that estimated the biomass of each cover crop species in three‐species and four‐species mixtures. The competition coefficients and models were validated with an additional nine site‐years testing the same cover crop mixtures. The biomass of a species in a site‐year was accurately predicted 69% of the time (low root mean square error, correlation > 0.5, not biased, *r* ^2^ > 0.5). Applying the framework, we designed three‐species and four‐species mixtures by identifying relative seeding rates that produced high biomass with high species evenness (i.e. high multifunctionality) at low seed costs based on a Pareto front analysis of 10,418 mixtures. Accounting for competition when constructing cover crop mixtures can improve the ecosystem services provided, and such an advancement is likely to lead to greater farmer adoption.

## Introduction

Cover crops can protect environmental quality, replace agricultural inputs with biological processes, and regenerate soil health (Teasdale 1996, Dabney and Delgado 2001, McDaniel and Tiemann 2014, Petit et al. 2018). Cover crops are not harvested but contribute to a more sustainable and multifunctional agro‐ecosystem by increasing the amount of species in a crop rotation (Lin et al. 2011, Davis et al. 2012, Gaudin et al. 2015, Bowles, 2020). As such, they provide temporal diversification and mixtures of cover crops can also provide spatial diversification. Temporal and spatial diversification reduces pest pressure of weeds (Liebman and Dyck 1993, Verret et al. 2017), insects (Tonhasca and Byrne 1994, Langellotto and Denno 2004), diseases (Boudreau 2013), and improves soil health (Vukicevich et al. 2016). Although cover crops may be used for singular purposes, cover crop mixtures can be used to achieve multiple benefits, as species perform different functions and can enhance the functions of other species. For example, legumes planted with grasses tend to fix nitrogen more efficiently (Brainard and Bellinder 2011, Schipanski and Drinkwater 2012), which results in a lower carbon to nitrogen ratio of the total biomass (Butler et al. 2012, Cong et al. 2015), and leads to faster N mineralization that could provide N to the subsequent crop or increase leaching potential.

Successfully establishing multiple species together at the same time can be difficult due to competition among plants for resources (Hall 1974). Competition within mixtures, as well as the ecosystem services they may deliver, is dependent on the growing conditions, management, and included species (Reiss and Drinkwater 2020). Asymmetric competition occurs when one or more species dominate due to particular traits that confer a fitness advantage (Funk and Wolf 2016) and suppress the growth of other species. Poorly constructed mixtures will not deliver the intended ecosystem services and are likely to impede adoption because the seed costs may be difficult to justify (Wayman et al. 2017, Roesch‐McNally et al. 2018, Bergtold et al. 2019).

**Fig. 1:**
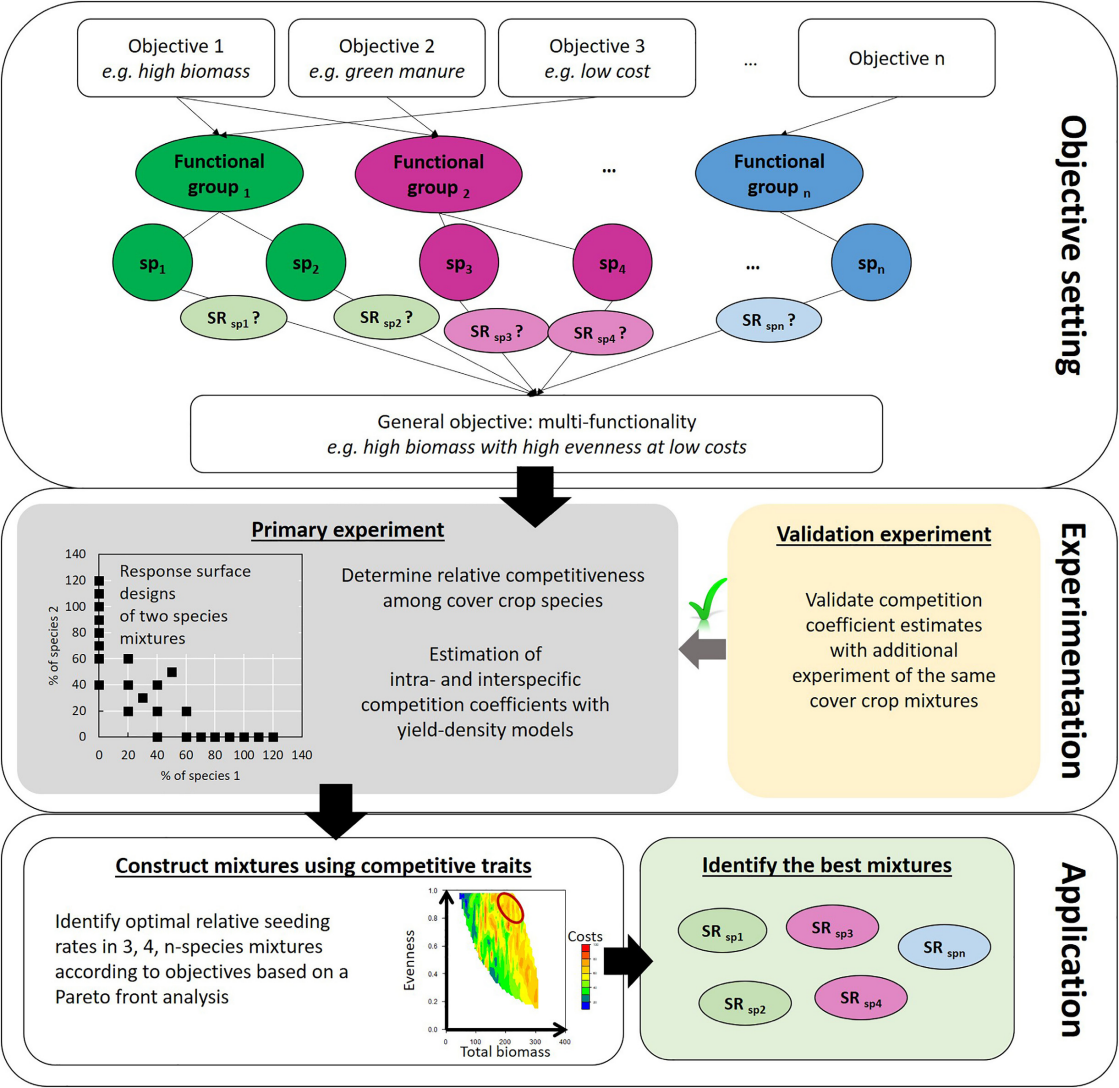
Framework for selecting cover crop mixture seeding rates.

We propose a framework to increase multifunctionality of mixtures while considering seed costs (Fig. 1). In it, crop biomass and species evenness of a cover crop mixture serve as coarse proxies for multifunctionality. Greater cover crop biomass tends to provide more ecosystem services, particularly weed suppression and reduced nitrate leaching (Schipanski, 2014). However, biomass alone is insufficient to provide multifunctionality (Smith and Atwood 2014). Previous studies (Finney et al. 2016 and Finney and Kaye, 2017), have shown that increasing functional diversity of cover crop mixtures is important for providing multiple ecosystem services. A high level of evenness means that each species present in a mixture produces a similar amount of biomass, indicating reduced asymmetric competition among species (Bybee‐Finley and Mirsky 2016). Computing biomass‐based evenness ensures that species of different sizes can perform their affiliated ecosystem service to a sufficient degree. By leveraging the seeding rates of each species in the mixture, competition can be managed and potential tradeoffs with overall biomass production and seed costs can be accounted for.

Practitioners typically use two approaches when constructing mixtures. The additive approach often uses monoculture seeding rates for each species in the mixture, resulting in a greater total density of plants and higher seed costs. The replacement approach often uses proportions based on a monoculture rate for each species divided by the number of species in the mixture. Replacement rates in intercropping research leads to findings that are highly conditional on the initial monoculture seeding rates used (e.g., Creamer and Baldwin 2000, Kadziulienė and Sarūnaitė 2011, Hayden and Ngouajio 2014) and confound intraspecific and interspecific competition as the seeding rates of the species are not changed independently.

An alternative approach uses a response surface design, which varies the densities of the two species separately and does not have the confounding effects that are found for additive or replacement rate designs. With this design, the monocultures of each species are planted at multiple densities and the bicultures are planted in multiple proportions. By applying a yield–density model to a response surface design the effects of intraspecific and interspecific competition and complementarity can be quantitatively determined (Spitters 1983).

Complementarity, the combined effects of resource partitioning and facilitation, is derived from combining species with different functional traits to access and use available resources more completely (Cardinale et al. 2011). The niche differentiation index (NDI) is a measure of complementarity determined by the ratio of overall intraspecific and interspecific competition in a biculture (Spitters 1983). An NDI above one indicates complementarity between two species, meaning that the net intraspecific competition is greater than the net interspecific competition. Niche differentiation between species in mixtures has been found in several experiments that use the Spitter's yield–density model and response surface designs: oat (*Avena sativa* L.) and faba bean (*Vicia faba* L.) bicultures (Helenius and Jokinen 1994), field pea (*Pisum sativum* L.) and oat bicultures (Neumann and Werner 2009), and all biculture combinations of mustard [*Brassica juncea* (L.) Czern.], field pea, black oat (*Avena strigosa* Schreb.) and phacelia (*Phacelia tanacetifolia* Benth.) (Wendling et al. 2017).

In this paper, we describe how our data‐driven approach can be applied to advance the multifunctionality of cover crop mixtures and provide greater precision to sustainable agriculture practices (Fig. 1). Our framework was developed using data from field research in the northeastern United States of four warm‐season cover crop species that represented two functional groups: grass and legume. Each species was grown as a monoculture and with every other species in biculture using a series of response surface designs, as well as in three‐species and four‐species polycultures using a replacement rate design. The relationship between seeding rate and crop performance was determined using a yield–density model to quantify intraspecific competition of each species in monoculture. An expanded yield–density model was used to quantify interspecific competition between species in each biculture. Competition coefficients from the biculture yield–density models were used to predict the biomass of each component species in three‐species and four‐species polycultures. Predicted biomass was compared with actual biomass using data from the three‐species and four‐species polycultures, and the approach was evaluated further using a separate dataset with nine site‐years. Generating 10,418 three‐and four‐species cover crop mixtures within the density limits of the experiment, the prediction equation simulated the biomass for each species, and the mixtures that best fit the multifunctional criteria were determined.

We hypothesized that (1) functionally diverse bicultures (i.e., grass–legume) will produce greater biomass and have greater niche differentiation compared with functionally similar bicultures (i.e. grass–grass and legume–legume), and (2) competition coefficients generated from bicultures can be used to accurately predict the biomass of three‐species and four‐species mixtures.

## Methods

The primary field experiment contained monocultures, bicultures, and three‐species and four‐species polycultures. It was conducted in 2014 at the Cornell Musgrave Research Farm in Aurora, NY (42°45′N, 76°35′W) on a Honeoye (fine‐loamy, mixed, semiactive, mesic Glossic Hapludalf) and Lima (fine‐loamy, mixed, semiactive, mesic Oxyaquic Hapludalf) silt loam, USDA plant hardiness zone 5b (minimum average temperature of −26.1°C to −23.3°C).

### Restricted response surface design using monoculture and biculture treatments

A series of restricted response surfaces was used to determine the effects of seeding rates on biomass production of four warm‐season annual cover crops grown in monocultures and each possible biculture combination (Table 1). Both the monocultures and the bicultures had eight different seeding rates. Two of the monoculture rates were greater than the recommended monoculture seeding rates to ensure that the intraspecific treatments measured maximum yield (i.e., asymptotic yield) for each species (Weiner and Freckleton 2010). Following this law of constant final yield (Weiner and Freckleton 2010), biculture seeding rate combinations were at or below replacement rate levels. Priority was given to the number of seeding rate treatments rather than replicates at a reduced number of rates to have a greater range of plant densities to better fill out the nonlinear regression model (Inouye 2001).

**Table 1:** Target seeding rates as a function of the recommended monoculture rates.

	Species 1	Species 2	Species 3	Species 4
Number of species in mixture	(Proportion of each species by its recommended monoculture seeding rate)			
1	0.4			
1	0.6			
1	0.7			
1	0.8			
1	0.9			
1	1			
1	1.1			
1	1.2			
2	0.2	0.2		
2	0.2	0.4		
2	0.2	0.6		
2	0.3	0.3		
2	0.4	0.2		
2	0.4	0.4		
2	0.5	0.5		
2	0.6	0.2		
3	0.33	0.33	0.33	
4	0.25	0.25	0.25	0.25

Notes

The four monocultures and six biculture combinations were each planted at eight rates. The five polyculture combinations were planted at one rate.

The four warm‐season annual crops tested were obtained from King’s Agriseeds (Ronks, PA) and included: pearl millet [*Pennisetum glaucum* (L.) R.Br.] (cv. ‘Wonderleaf’), sorghum sudangrass [*Sorghum bicolor* (L.) Moench × *Sorghum sudanense* (Piper) Stapf], (cv. ‘AS6401’), sunn hemp (*Crotalaria juncea* L.) (VNS), and cowpea [*Vigna unguiculata* (L.) Walp] (cv. ‘Iron and Clay’). These species used as cover crops can produce large amounts of biomass, are drought‐tolerant, and can be used as a forage crop, or in small grains or vegetable production systems after a spring crop is harvested and before a fall crop. Both grasses are C_4_ and native to northeastern Africa (Andrews and Rajewski 1993). Pearl millet is a deep‐rooted cereal grain with a high tillering ability and can reach 1–2.5 m (Newman and Jennings 2010, Lee et al. 2012, Sheahan 2014). Pearl millet matures earlier than sorghum [*S. bicolor* (L.) Moench] and is more drought‐tolerant (Jefferson Institute 2002). Sorghum sudangrass is a cross between sorghum and sudangrass [*S. sudanense* (Piper) Stapf] that provides the yield advantage of sorghum with the finer stem of sudangrass and can reach 1.2–4 m tall. It also has the ability to tiller. The sorghum sudangrass variety used in our research had a brown‐midrib mutation that reduces the lignin content and provides higher forage quality (Miller and Stroup 2003). Sunn hemp is a fibrous legume native to India (Cook and White 1996). Sunn hemp can grow 1–3 m and has leaves radiating around the stem (NRCS 1999, Sarkar et al. 2015). Cowpea is an herbaceous, tap‐rooted legume native to western Africa (Davis et al. 1991). It does not grow well in alkaline or poorly drained soils (SARE 2012), but is shade tolerant (Blade et al. 1997). Iron and Clay is a blend of two cultivars: one with a upright bushy growth habit, while the other has a vining, prostrate growth habit.

The recommended seeding rates for each cover crop species (and abbreviations used from this point forward) in monoculture were: pearl millet (M) 2.1 g seed m^−2^ (292 seeds m^−2^), sorghum sudangrass (S) 6.6 g seed m^−2^ (218 seeds m^−2^), sunn hemp (H) 5.6 g seed m^−2^ (146 seeds m^−2^), cowpea (C) 7.0 g seed m^−2^ (72 seeds m^−2^). The rates used as a basis (100% recommended seeding rate) were within the recommended range for the species according to the seed supplier. Pearl millet, sunn hemp, and cowpea had germination rates of 80% and sorghum sudangrass had a germination rate of 85% according to the seed label. To ensure consistent targeted emergence rates among species, seeding rates based on seed weights were adjusted for a live‐seed basis. Seed costs per kg were US$3.13, 3.66, 5.69, and 4.52 for M, S, H, and C, respectively. These costs are within range for typical costs for producers, particularly those who are growing higher value vegetables.

### Replicated three‐species and four‐species polycultures

A factorial design of each possible three‐species and four‐species mixture using the same species was replicated five times. To do this, we used replacement seeding rates such that each species included in the polyculture was seeded at the recommended seeding rate of the monoculture divided by the number of species in the polyculture (Table 1). The treatments in the restricted response surface design and the factorial design were randomized in the field to control for spatial heterogeneity.

### Management

Preceding this experiment, the field contained conventional corn in 2012, conventional soybeans in 2013, and then planted into conventional winter wheat in the fall of 2013, it was sprayed with glyphosate in the spring of 2014. Afterwards, the field was chisel plowed, disked, and cultipacked prior to planting the cover crops. Then, the primary experiment was managed organically. Before cover crop planting, 35 kg ha^−1^ of total N was broadcast in the form of poultry manure (5–4–3). Although this is more N than would typically be applied before legume crops, it represents less than half of the recommended rate for sorghum sudangrass (Ketterings et al. 2007). The legumes were inoculated with the N‐Dure rhizobium mixture (Verdesian Life Sciences, Cary, NC, USA) suitable for cowpea and sunn hemp before planting.

Cover crops were planted on July 7, 2014 to a depth of approximately 2.5 cm in 18 cm rows with six rows using a custom‐built cone seeder drill with hydraulic twin disk row openers and packing wheels. Plots measured 1.26 m by 6 m with a buffer of 1.5 m between plots maintained by mowing to prevent potential light competition between plots.

### Sampling

Crop and weed biomass samples were collected 50 d after planting (DAP; 473 growing degree days, base temperature 10°C). In two 0.5 m^2^ quadrats centered in the plot, plants were clipped 10 cm above the soil surface, separated by species, dried at 60°C for at least 1 week, and then weighed. Within each quadrat, cover crop and weed density were counted by species at the same time as the biomass sampling. Individual plants of grass species were distinguished by tracing stems to the soil to avoid counting tillers as separate plants.

### Data analysis

Data were analyzed using R 3.3.2 (R version 3.3.2, Inc., Boston, MA, 2015). Analyses were performed using the observed densities unless otherwise stated, as the seeding densities differed somewhat from the observed densities of emerged plants (Appendix S1: Fig. S1).

To summarize the results, three separate Type III analyses of variance tests were used to compare total cover crop biomass among (1) the four monoculture treatments (*n* = 8 for each of the cover crop monocultures), (2) the six biculture treatments (*n* = 8 for each of the cover crop bicultures), and (3) the five polyculture treatments (*n* = 4 for each of the polycultures). As the monoculture and bicultural treatments were not replicated, seeding rate levels were used as replicates for both the monoculture and biculture treatments, assuming no differential responses to seeding rates for the purpose of presenting an initial sense of seed treatment effects. Tukey's honest significance difference test was performed to compare treatment means at α = 0.05. Pearl millet had only seven monoculture treatments, as density was not recorded in the 60% seeding rate treatment.

### Yield–density competition model

Crop biomass and density data were used to quantify intraspecific and interspecific competition using data from the restricted response surfaces (Willey and Heath 1969). Using the eight seeding rates of a species in monoculture, *Y*_1_ is the biomass of species 1, the focal species, calculated using (Eq. 1):

(1) $$Y_{1}=\frac{N_{1}}{b_{1,0}+b_{1,1}N_{1}}$$

The term, *N*_1_ is the density of the species *I*. The term, *b*_1,0_ is necessary to ensure that when *N*_1_ = 0, *b*_1,1_ = 0, *Y*_1_ = 0 and when *N*_1_ = 1, *b*_1,1_ = 0, Y_1_ ≠ 0. The term is a more influential parameter when density is low. The intraspecific competition coefficient, *b*_1,1_ (m^2^ g^−1^), describes how the per plant weight decreases when additional plants of the same species are added. To determine the intraspecific competition coefficient for each species in monoculture, yield–density models (Eq. 1) were fitted using nonlinear regression to the observed density and biomass for each species in monoculture.

The yield–density model was expanded for a biculture to account for interspecific competition with the assumption that additional species hold a similar hyperbolic relationship (Spitters 1983). In which *Y*_1,2_ is the biomass of species 1 in a biculture of species 1 and 2 and was calculated using (Eq. 2):

(2) $$Y_{1,2}=\frac{N_{1}}{b_{1,0}+b_{1,1}N_{1}+b_{1,2}N_{2}}$$

where *N*_1_ and *N*_2_ are the plant densities of each species. The interspecific competition coefficient, *b*_1,2_ (m^2^ g^−1^), is a measure of interspecific competition of species 2 on species 1 and describes how the per plant weight of species 1 decreases when plants of species 2 are added. The relationship can be made linear by using the inverse of the per plant weight, as yield (*Y*_1_) equals the weight per plant times the density (*N*_1_) (Appendix S1: Eq. S1). As a visual aid to help the reader understand how the shape of the yield–density curve changes under different competition scenarios, examples using various competition coefficients can be found in the appendix (Appendix S1: Fig. S2). For the bicultures, intraspecific and interspecific competition coefficients were determined using the expanded yield–density model (Eq. 2). Models were fitted using nonlinear regression to the observed density and biomass of the response surface design (combined monoculture and biculture data) for each species in each biculture.

Initially, weed density was included as a term in the model but resulted in a higher Akaike Index Criterion (AIC) without improving the model fit, so the weed density term was omitted. Briefly, weed biomass was generally low with an average of 16 weeds per square metre weighing 6 g m^−2^. Several plots had no weeds (cowpea seeded at 110%, pearl millet–cowpea seeded at 40% and 20% respectively, and one replicate of the pearl millet–sorghum sudangrass–cowpea mixture). The bicultures of pearl millet‐cowpea seeded at 20% and 40%, respectively, and sunn hemp–cowpea each seeded at 40% had the greatest amount of weeds (40 plants m^−2^ with 89 g and 46 plants m^−2^ with 16.1 g, respectively). Weed biomass tended to be lower in plots with high levels of cover crop biomass (data not shown). The most common weed species was Venice mallow (*Hibiscus trionum* L.), a legacy from a previous experiment.

### Theoretical maximum weight and maximum yield

Previous work on yield–density models has not described the relevance of the reciprocal intercept term, 1/*b*_1,0_ (g per plant). Spitters (1983) described it as the virtual biomass of an isolated plant. The term describes the theoretical maximum weight per plant as a function of the growing conditions and portrays the potential of a plant, garnering an idea of what may be possible if competition is managed in a mixture. Similarly, the reciprocal of the intraspecific competition term, 1/*b*_1,1_ (g m^−2^), represents the theoretical maximum yield.

### Assessing competition and complementarity between species

Competition in bicultures was assessed by comparing intraspecific and interspecific competition coefficients. These were first normalized to control for size bias by dividing the competition coefficients by the intercept (*b*_1,1_/*b*_1,0_ and *b*_1,2_/*b*_1,0_), as an increase in the density of a larger plant results in a greater biomass than that of a smaller species (Neumann et al. 2009). Two indices were used to measure the influence of species interactions on mixture performance. The relative competitive ability (RC) is the ratio of intraspecific competition to interspecific competition independent of the density of either species in the biculture (Eq. 3). Biologically, it is the number of plants of species 1 that can replace plants of species 2 without changing the per plant weight of species 1 (Helenius and Jokinen 1994):

(3) $$\text{RC}=\frac{b_{1,1}}{b_{1,2}}$$

The NDI uses the four competition coefficients of a biculture to assess resource complementarity (Eq. 4). It is the product of both species RCs:

(4) $$\text{NDI}=\frac{b_{1,1}}{b_{1,2}}\times \frac{b_{2,2}}{b_{2,1}}$$

### Predicting crop biomass of three‐species and four‐species polycultures

Intercepts and competition coefficients determined with response surface data were used to predict biomass for each species in three‐species and four‐species polycultures (Eq. 5). The model is specific to species composition of the polyculture (e.g. called 1, 2, 3,…, *r*) with yield of a focal species *a* in the polyculture, calculated by:

(5) $$Y_{a\text{in}1,2,3,\ldots ,r}=\frac{N_{a}}{{\bar{b}}_{a,0}+{\bar{b}}_{a,a}N_{a}+\sum_{i\text{in}\left\{N\backslash \left\{a\right\}\right\}}^{r}b_{a,i}N_{i}}$$

where ${\bar{b}}_{a,0}$ and ${\bar{b}}_{a,a}$ are means of $b_{a,0}$ intercept coefficients and $b_{a,a}$ intraspecific competition coefficients of species *a* in each *a*,*i* bicultures of a mixture containing *r* species, where $\left\{N\backslash \left\{a\right\}\right\}$ means the *i* term cannot be *a* (i.e., in the millet–sorghum sudangrass–sunn hemp (MSH) polyculture where *a* is pearl millet, M, estimates are based on millet–sorghum sudangrass (MS) and millet–sunn hemp (MH) bicultures intercepts and competition coefficients). Here, we continued with the assumption made by Spitters (1983) that indirect competition from additional species (i.e., interactive effects among species) is quite small compared with direct competition, and therefore we do not account for it.

To determine if the competition model (Eq. 5) fitted one species better than another, the observed and predicted biomass were first scaled using *Z*‐score normalization (μ = 0 and σ = 1) by species to account for magnitudes of difference in biomass. Using the scaled predicted and observed data, the fits of the models were assessed with four indicators: (1) the root mean square error (RMSE) to describe the difference between the predicted and observed values, (2) the coefficient of correlation (slope) of a simple linear regression between the predicted and observed biomass to describe the strength of the relationship, (3) a test (slope ≠ 1) to determine if the model exhibited bias, and (4) the coefficient of determination (*r* ^2^) to describe the strength of the correlation (Mudrak et al. 2014).

### Model validation

The results from a two‐year summer cover crop experiment using the same polycultures and biomass sampling methods (called from this point forwards the validation experiment) were used to test the ability of our model to make accurate predictions of crop biomass of three‐species and four‐species polycultures across different growing conditions. The validation experiments were conducted in 2013 and 2014 at: (1) the Cornell Musgrave Research Farm (Aur) on the same soil types as the primary experiment; (2) the Cornell Willsboro Research Farm (Wil) in Willsboro, NY (44°21′N, 73°23′W) on a Stafford fine sandy loam (mixed, mesic Typic Psammaquent), plant hardiness zone 4b (minimum average temperature of −31.7° to −28.9°C); and (3) the USDA‐ARS Beltsville Agricultural Research Center (Bel) in Beltsville, MD (39°02′N, 76°54′W) on an Elkton silt loam (fine‐silty, mixed, active, mesic Typic Endoaquult), plant hardiness zone 7a (minimum average temperature of −17.8° to −15°C). The Bel location included two different field sites in 2013, and the Aur and Wil locations included two different field sites in 2014. Different field sites within a location were planted 1 month apart, as indicated by “E” (for early) and “L” (for late). Therefore, the validation experiment had nine site‐years. A randomized complete block design was used (except for the BelL13 site‐year) with four to five blocks per site‐year for a total of 199 plots. Biomass was sampled from 43 to 53 DAP using the same protocol described in *Sampling*. Slight modifications to management practices, planting dates, and plot sizes across sites and years occurred (see Bybee‐Finley et al. 2016 for more details).

The competition model (Eq. 5) was run using the observed densities from the experiment to predict the crop biomass of a focal species. Observed and predicted data were scaled using *Z*‐score normalization (μ = 0 and σ = 1) by site‐year and species to account for species size and control for the variety of growing conditions. The normalized data were assessed using the same four steps described above.

### Pareto front to determine optimal three‐species and four‐species polycultures

To find the optimum densities of species for achieving high total biomass and high species evenness in three‐species and four‐species polycultures, the competition models (Eq. 5) were used to compute the biomass for each focal species of a simulated mixture. Simulated mixtures were generated so that they fit within the limits of the model, i.e., the seeding rates of the simulations ranged from 0 to 120% of the recommended monoculture seeding rates (i.e. from 0 to 350, 262, 176, and 86 seeds m^−^² for pearl millet, sorghum sudangrass, sunn hemp, and cowpea, respectively). To do this, increments proportional to 5% of the maximum seeding rates of each species (increments of 17, 13, 8, 4 seeds m^−2^ respectively for pearl millet, sorghum sudangrass, sunn hemp and cowpea) were used. To respect the limits of the model, mixtures were excluded when the sum of the proportions of each species exceeded 120%, so that biomass of each species for 10,418 mixtures was computed.

The total biomass was the sum of each species biomass. The species evenness based on biomass was computed using the *vegan* package (Oksanen et al. 2020) (Eq. 6):

(6) $${\text{Pielou}}^{'}\text{s species evenness}=\frac{‐\sum_{i=1}^{S}{\text{pi}}_{1}\ln\left(\text{pi}\right)}{\ln\left(S\right)}$$

where the numerator is the Shannon diversity index, pi is the proportion of biomass belonging to the *i*th species, and *S* is the number of species in the mix. The denominator is the maximum Shannon diversity, ln(*S*). Species evenness is bounded between 0 and 1, with increasing evenness until the maximum of 1 is reached, meaning equal amount of biomass for each species in the intercrop. Mixture costs, based on the cost of seeds at time of purchase and accounting for germination rate, were also computed. The output was plotted using contour plots with the *Akima* package (Akima et al. 2016).

To find the best mix, we identified “Pareto‐optimal” mixtures following the methodology developed by Lafond et al. (2017). The Pareto front method identifies “efficient” and “non‐dominated” solutions that perform at least as well as others for all criteria and strictly better for at least one criterion (Kennedy and Ford 2011). This set of solutions is also called the “Pareto‐optimal set” and forms the “Pareto front,” on which it is not possible to increase a criterion without reducing at least another one. Here, we identified the Pareto‐optimal set of mixtures that optimized total biomass and species evenness, followed by consideration of seed cost for the best mixtures. From the Pareto front, a subset of mixtures was selected that had a species evenness greater than 0.8. Although little research has been done regarding a desirable level of species evenness in cover crop mixtures, Tracy and Sanderson (2004) reported that higher levels of evenness (0.8–1.0) resulted in lower weed abundance in a survey of 37 pastures in the northeastern USA.

## RESULTS

### Cover crop production in primary experiment

#### Monoculture treatments

**Fig. 2:**
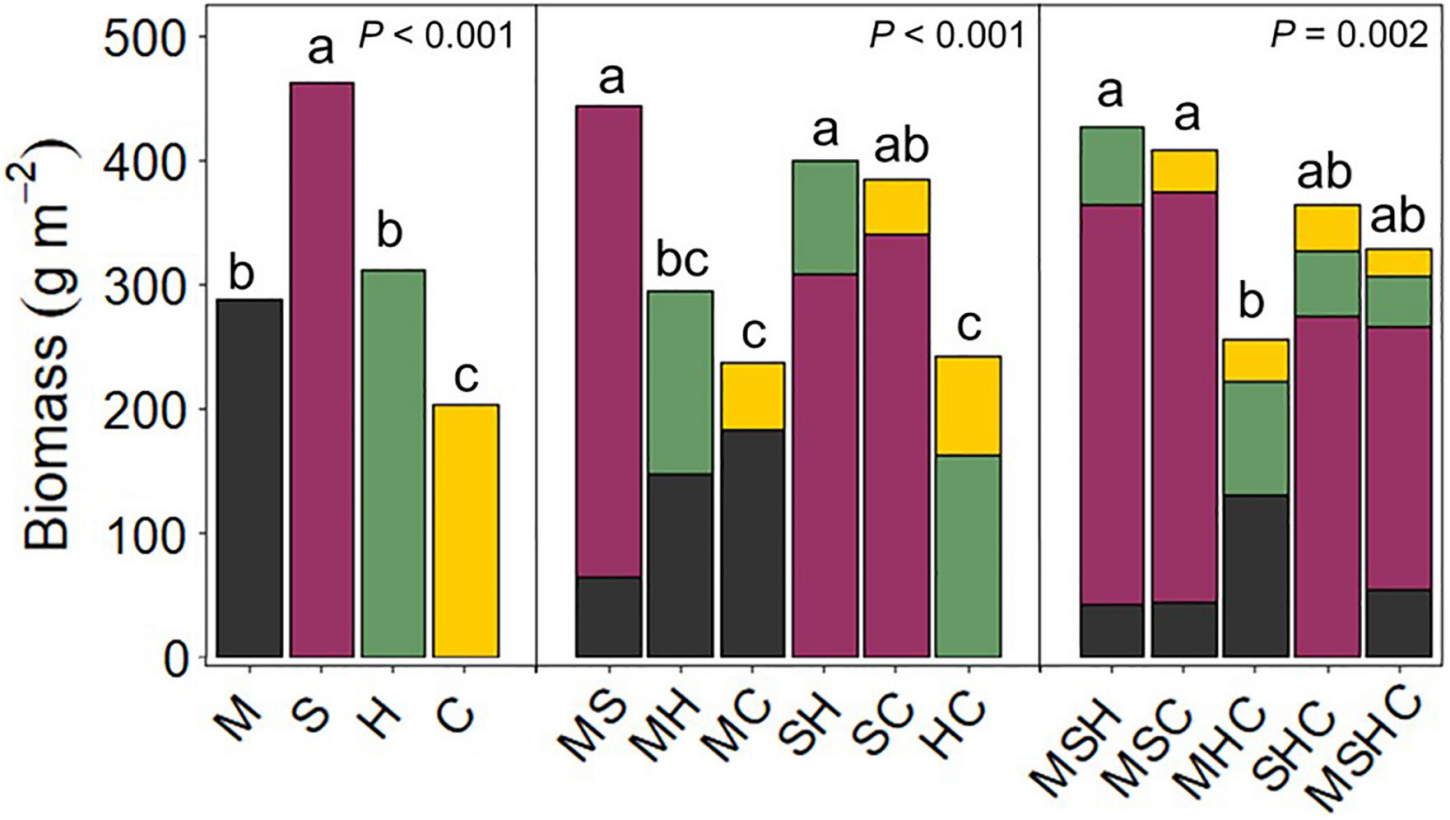
Analysis of variance of average crop biomass across mixture treatments. Analyses of variance (ANOVAs) were performed based on species richness, such that monocultures, bicultures, and polycultures were assessed separately. Similar letters above bars within a panel (e.g., monoculture, biculture, and polyculture) indicate no significant differences between treatments in total crop biomass at *P* < 0.05. C, cowpea; H, sunn hemp; M, pearl millet; S, sorghum sudangrass.

Sorghum sudangrass produced the greatest amount of biomass in monoculture (Fig. 2) with an average of 462 g m^−2^ and a range of 398–511 g m^−2^, which was observed in the 70% and 60% seeding rate treatments, respectively. Pearl millet and sunn hemp monocultures produced similar amounts of biomass with averages of 287 and 312 g m^−2^, respectively. Pearl millet biomass ranged from 254 to 366 g m^−2^ at the 100% and 90% seeding rates, respectively. Sunn hemp biomass ranged from 263 to 344 g m^−2^ at the 40% and 90% seeding rates, respectively. The cowpea monoculture produced the least amount of biomass, resulting in an average of 203 g m^−2^, less than half the amount of the sorghum sudangrass monoculture. Cowpea biomass ranged from 171 to 246 g m^−2^ at the 40% and 110% seeding rates, respectively.

#### Biculture treatments

The treatments that contained sorghum sudangrass were the most productive bicultures (Fig. 2). The sorghum sudangrass–cowpea (SC) biculture produced similar amounts of biomass as the pearl MS and the sorghum sudangrass–sunn hemp (SH) bicultures but more than the other bicultures containing cowpea. The pearl MH biculture produced a similar amount of biomass as the bicultures containing cowpea. Biomass of each species in the bicultures was lower than their biomass in monocultures, which was expected. Higher seeding rates typically resulted in more biomass. However, the extent of the reduction of biomass in biculture compared with monocultures differed by species as a result of asymmetric competition.

#### Polyculture treatments

As with the bicultures, including sorghum sudangrass in a polyculture generally resulted in greater total biomass compared with polycultures without sorghum sudangrass. However, no transgressive overyielding was observed in the polycultures, i.e., no polyculture yielded more than the most productive monoculture. Within the polycultures, sorghum sudangrass constituted the majority of the biomass and suppressed the growth of the other included species. The pearl millet–sunn hemp–cowpea (MHC) polyculture was less productive than the pearl millet–sorghum sudangrass–cowpea (MSC) and the pearl MSH polycultures (Fig. 2). The coefficients of variation (CV), found by dividing the standard deviation of a polyculture by its mean biomass, were 0.19, 0.20, 0.18, 0.12, and 0.12 for MSH, MSC, MHC, sorghum sudangrass–sunn hemp–cowpea (SHC), and pearl millet–sorghum sudangrass–sunn hemp–cowpea (MSHC), respectively. The SHC and MSHC polycultures, which contained sorghum sudangrass and the two legumes, had the most stable production.

#### Intraspecific competition and maximum yield of the monocultures

Intraspecific competition was greatest in sorghum sudangrass, followed by cowpea, pearl millet, and sunn hemp (Table 2, raw numbers are listed in Appendix S1: Table S1). The competition coefficient for sunn hemp was less than half that of the competition coefficients for sorghum sudangrass and cowpea. Maximum yield (1/*b*_1,1_) occurs at the asymptote and therefore at an infinitely high density. Sorghum sudangrass had the largest maximum yield, followed by sunn hemp, pearl millet, and cowpea (540, 470, 357, and 302 g m^−2^, respectively).

**Table 2:** Parameter values and competition indices of the four monocultures and six bicultures determined by the yield–density model.

Treatments	Species	*b*_1,0_ (plant g^−1^)	*b*_1,1_/*b*_1,0_ (m^2^ plant^−1^)	*b*_1,2_/*b*_1,0_ (m^2^ plant^−1^)	RC	NDI	*r* ^2^
M	M	0.1336	0.0210				0.19
S	S	0.0491	0.0377				0.66
H	H	0.1606	0.0132				0.44
C	C	0.0930	0.0356				0.86
MS	M	0.1319	0.0213	0.0622	0.34	3.62	0.75
MS	S	0.0658	0.0266	0.0025	10.57		0.59
MH	M	0.1646	0.0161	0.0175	0.92	1.35	0.83
MH	H	0.2091	0.0088	0.0060	1.47		0.9
MC	M	0.1920	0.0134	0.0041	3.27	3.2	0.46
MC	C	0.0970	0.0335	0.0342	0.98		0.94
SH	S	0.0839	0.0203	0.0078	2.59	1.31	0.57
SH	H	0.1502	0.0146	0.0289	0.51		0.95
SC	S	0.0644	0.0279	0.0226	1.23	0.95	0.24
SC	C	0.1007	0.0317	0.0412	0.77		0.78
HC	H	0.1548	0.0141	0.0106	1.32	1.27	0.89
HC	C	0.0931	0.0355	0.0369	0.96		0.97

Notes

The data used in the expanded yield–density model for the bicultures contained the focal species in monoculture and in biculture to determine intraspecific and interspecific competition coefficients. The term *b*_1,0_ is the intercept; *b*_1,1_, intraspecific competition coefficient; *b*_1,2_, interspecific competition coefficient; RC, relative competitive ability; NDI, niche differentiation index; *r* ^2^ of observed and predicted biomass standardized by species (μ = 0 and σ = 1); M, pearl millet; S, sorghum sudangrass; H, sunn hemp; C, cowpea.

### Competitive dynamics of the restricted response surface

#### Intraspecific and interspecific competition within the response surfaces

**Fig. 3:**
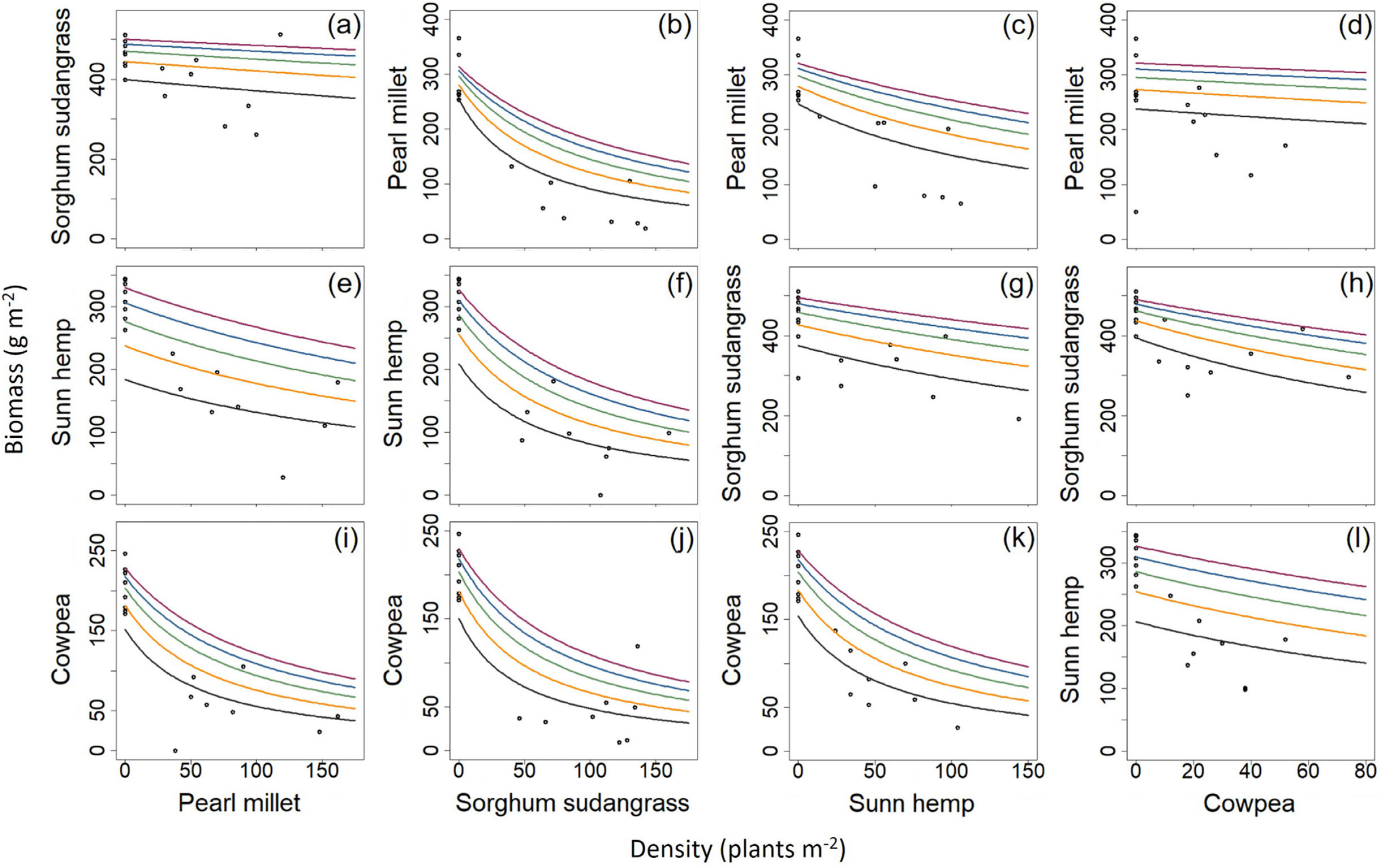
Each panel (a–l) depicts the biomass of the focal species and the density of the other species in the biculture with each row representing a different biculture. The data used in the expanded yield–density model contained the focal species in monoculture and in biculture to determine intraspecific and interspecific competition coefficients. The points at zero density of a species represent the biomass of the focal species when planted in monoculture. The different lines represent different proportions of the recommended monoculture seeding rate of the focal species, illustrating the effects of increasing the density of the non‐focal species.

Pearl millet had the most variable response to the other species in the biculture. Pearl millet plants faced the highest level of interspecific competition from sorghum sudangrass and the lowest level of interspecific competition from cowpea (0.0622 and 0.0134 m^2^ per plant, respectively, Table 2). As such, pearl millet biomass declined sharply with increasing sorghum sudangrass density but only slightly with increasing cowpea density (Fig. 3b, d). Sorghum sudangrass produced a large amount of biomass even at low seeding rates. Increasing the densities of pearl millet, sunn hemp, and cowpea had slight negative effects on the sorghum sudangrass biomass (Fig. 3a, g, h). More than the other two species, increasing the density of cowpea led to greater decline of sorghum sudangrass biomass (Fig. 3h). Sunn hemp faced the highest level of interspecific competition from sorghum sudangrass and the lowest level of interspecific competition from pearl millet (0.0289 and 0.0060 m^2^ per plant, respectively, Table 2). For this reason, sunn hemp biomass declined sharply with increasing sorghum sudangrass density but at a slower rate when increasing pearl millet density (Fig. 3f and e). Cowpea biomass declined sharply with increasing pearl millet and sorghum sudangrass density (Fig. 3i and j), but less so with sunn hemp (Fig. 3k). Compared with its other bicultures, cowpea had the lowest intraspecific competition but faced the most interspecific competition when in biculture with sorghum sudangrass (Table 2).

Overall, sunn hemp generally had the lowest level of intraspecific competition and cowpea had the greatest, followed by sorghum sudangrass (Table 2). Cowpea faced the greatest level of intraspecific competition of any species in biculture when it was paired with sunn hemp (0.0355 m^2^ per plant; Table 2). The competitive dynamics of pearl MS and sunn hemp–cowpea (HC) (Table 2) show that intraspecific competition of a less competitive species (pearl millet and cowpea, respectively) is the greatest when the biculture is composed of two species in the same functional group (i.e., grass–grass, legume–legume).

We observed that competitive properties are intransitive. Of all the bicultures, pearl millet faced the greatest amount of interspecific competition from sorghum sudangrass. Cowpea in all three of its bicultures faced the next greatest levels of interspecific competition. Cowpea affected pearl millet very little, yet cowpea affected sorghum sudangrass more than any other species (interspecific competition coefficient of 0.0041 and 0.0226 m^2^ per plant, respectively; Table 2).

### Relative competitive ability of each species

Despite being functionally similar, the grasses had very different patterns in the ratio of interspecific to intraspecific competition. In the bicultures, the intraspecific competition faced by pearl millet was lower than the interspecific competition from sorghum sudangrass and sunn hemp, resulting in RCs of less than one. In contrast, sorghum sudangrass had higher levels of intraspecific competition and lower levels of interspecific competition, resulting in RCs consistently above one. All species planted with sorghum sudangrass had RCs less than one, indicating that they faced greater interspecific competition than intraspecific competition (Table 2). The biculture of the two grasses, had the greatest difference of RCs. In the pearl MS biculture, the RC of pearl millet was 0.34, meaning that the pearl millet plants could sense the presence of one pearl millet plant as strongly as the presence of 0.34 sorghum sudangrass plants (Table 2). In the pearl millet–cowpea (MC) biculture, the intraspecific competition of pearl millet was much higher than interspecific competition from cowpea, resulting in an RC of 3.27 (Table 2). The RCs of pearl millet in the pearl MH biculture, cowpea in the pearl MC biculture, and cowpea in the sunn hemp–cowpea (HC) biculture were close to one (Table 2), indicating similar levels of intraspecific and interspecific competition of species in bicultures. Cowpea had high levels of both intraspecific and interspecific competition indicating that cowpea performs best when planted at lower seeding rates. By examining whether intraspecific or interspecific competition was higher for each species in each biculture, the following hierarchy from most to least competitive was established: sorghum sudangrass > sunn hemp > pearl millet > cowpea.

### Niche differentiation

All bicultures had NDIs greater than one, apart from one, indicating that in most cases species exhibited complementarity. The NDI for MS was large because of the low intraspecific competition that pearl millet had with itself and low level of interspecific competition it gave to the sorghum sudangrass (Table 2). Whereas, the large NDI for MC was because the species faced greater overall intraspecific competition than interspecific competition, particularly pearl millet faced low interspecific competition from cowpea. The SC biculture had the only NDI below one because the overall interspecific competition was greater than the intraspecific competition that either species faced.

As documented in Wendling et al. (2017), a positive correlation was observed between total biomass of the biculture treatments and NDI (Kendall's coefficient of correlation τ = 0.20, *P* = 0.04). The bicultures that contained sorghum sudangrass generally produced more biomass than those that did not, therefore more biomass was produced when the most productive species was included. More biomass was produced by bicultures that faced lower overall interspecific competition, but they were not necessarily functionally diverse.

### Theoretical maximum plant weight

Intercept (*b*_1,0_) values were variable with larger standard errors compared with the other model parameters (Appendix S1: Table S1). The theoretical maximum weight of a plant (1/*b*_1,0_) changed from when species were planted in monoculture or biculture, sometimes increasing, which indicated the potential benefits of growing species together. In monocultures, the theoretical weights were 20.4, 10.8, 7.5, and 6.2 g per plant for sorghum sudangrass, cowpea, pearl millet, and respectively.

The theoretical maximum weight of sorghum sudangrass changed the most when it was planted with another species, dropping to 11.9 g per plant when in biculture with sunn hemp. The theoretical weight of sunn hemp was largest when it was planted with sorghum sudangrass (6.7 g per plant) and smallest when it was planted with pearl millet (4.8 g per plant). The theoretical weight of pearl millet was largest with sorghum sudangrass (7.6 g per plant). In an MC biculture, cowpea (10.31 g per plant) was theoretically a larger plant than pearl millet (5.21 g per plant), but increasing the seeding rate of pearl millet led to a greater increase in biomass because the pearl millet sowing density was higher and it faced less intraspecific competition than that of cowpea.

### Predicting crop biomass in polycultures in primary experiment

**Fig. 4:**
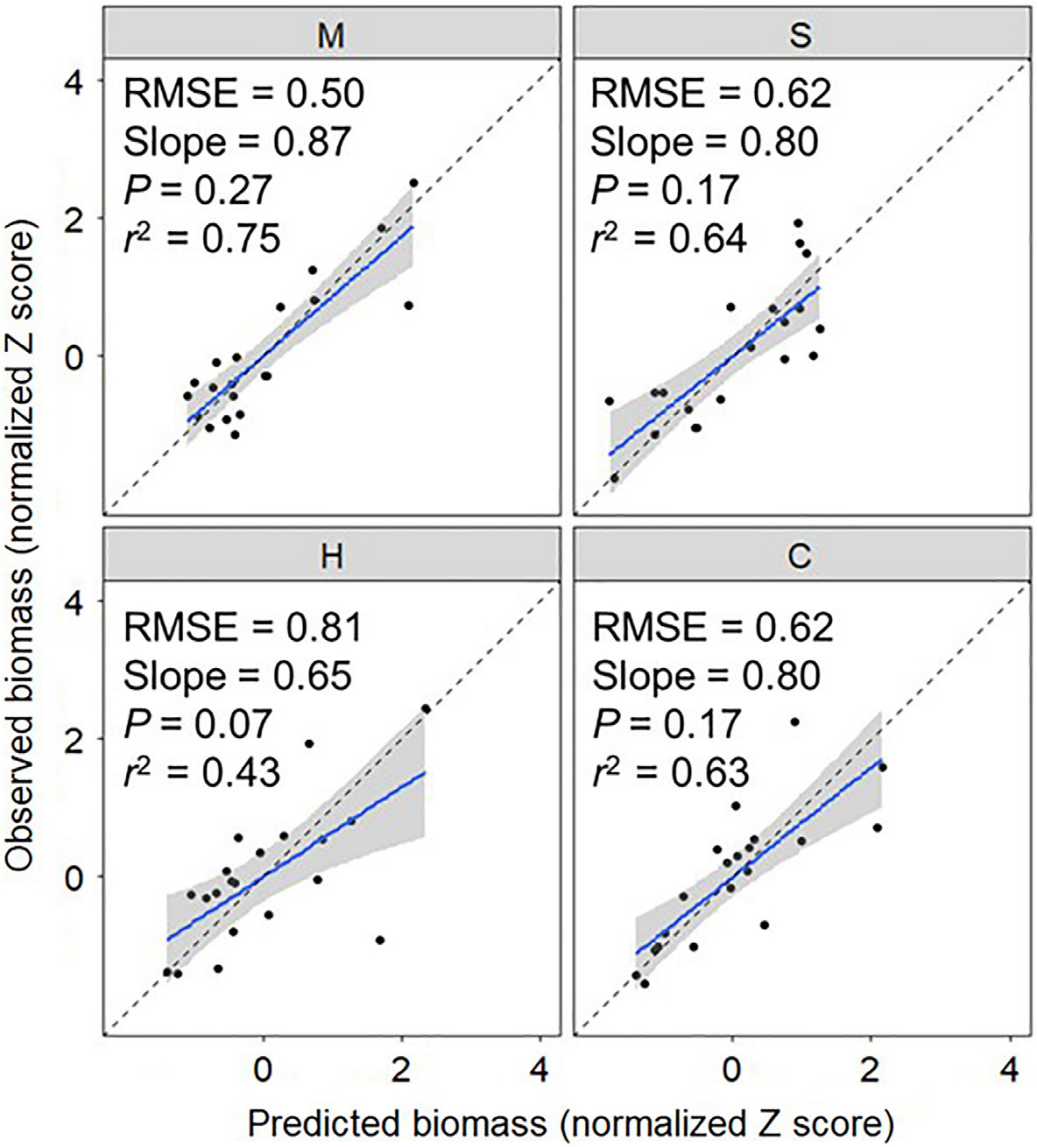
The fit of the predicted biomass from the models that used the competition coefficients from the response surface designs to the observed biomass for each species in the polycultures. The blue line reflects a linear regression between the observed and predicted biomass scaled by species. The dotted line shows Observed = Predicted, reflecting a perfect prediction. The shaded area represents the 95% confidence interval. C, cowpea; H, sunn hemp; M, pearl millet; RMSE, root mean square error; S, sorghum sudangrass; slope, the coefficient of correlation between observed and predicted; *P*‐value (slope ≠ 1) describes if the model predictions had bias; *r* ^2^, the coefficient of determination describes the strength of the correlation.

As the competition coefficients used were generated under the same growing conditions as the polycultures, we demonstrated the ability of Eq. 5 to accurately predict crop biomass in three‐species or four‐species polycultures (Fig. 4). Models fit each species well and the predicted biomass values were similar to the observed biomass of the polycultures. Models fit pearl millet the best and sunn hemp the worst. No bias was detected in the model predictions.

### Predicting crop biomass in validation experiment

**Fig. 5:**
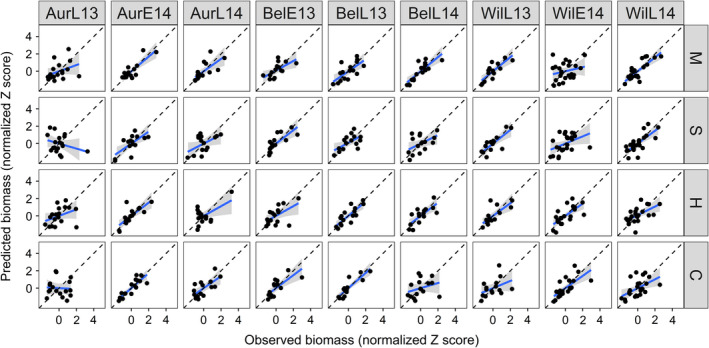
Visualization of model validation using observed biomass from nine site‐years of data, which took place in three locations over two years, in which some sites had experiment replicates. Data are standardized for each species within each site (μ = 0 and σ = 1). The blue line reflects a linear regression and the dotted line shows Observed = Predicted, reflecting a perfect prediction of the validation data. The shaded area represents the 95% confidence interval. Aur, Aurora, NY; Wil, Willsboro, NY; Bel, Beltsville, MD; “E” and “L” correspond to early and late planting date; 13 and 14 correspond to planting date year. C, cowpea; H, sunn hemp; M, pearl millet; S, sorghum sudangrass.

The models successfully predicted the crop biomass in 5, 7, 7, and 6 of the site‐years for cowpea, sunn hemp, millet, and sorghum sudangrass, respectively (total, 25/36 = 69%) (Fig. 5; Appendix S1: Table S2). In four out of nine site‐years, the model successfully predicted biomass of all four species. Across site‐years, the model best predicted pearl millet biomass, followed by sunn hemp, sorghum sudangrass, and finally cowpea. This is likely to be due to the competition coefficients generated in the response surface designs better representing pearl millet across growing conditions than cowpea.

Across the site‐years, no species‐specific trends in model fitness emerged. The model performed the best in the AurE14, BelE13, and BelL13 site‐years. Interestingly, in the one site‐year that used synthetic fertilizer (AurL13) instead of poultry manure, the model did not predict crop biomass for any species well (higher RMSEs, lower slopes and *r* ^2^ values, and bias in predictions). This is a clear indication of changes in competition dynamics under different management practices. If only site‐years with similar fertilizer regimes were included, the model accurately predicted crop biomass 78% of the time. In the WilE14 site‐year, the model did not predict the grass species well, and in the WilL14 site‐year, the model did not predict the legume species well. The model tended to overpredict biomass production of all species, especially the grasses, but these were within the confidence intervals.

### Optimal mixtures

**Fig. 6:**
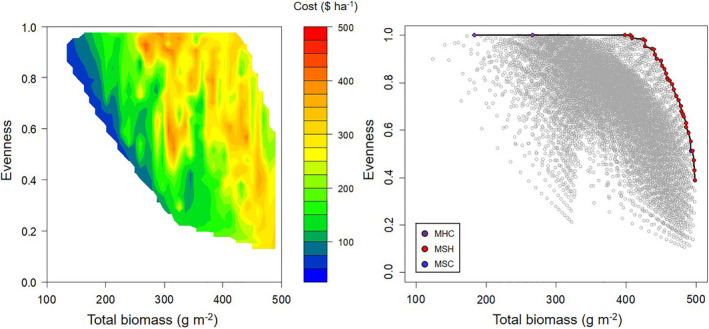
The total biomass and species evenness of three‐species and four‐species mixtures (*N* = 10,418) of pearl millet (M), sorghum sudangrass (S), sunn hemp (H) and cowpea (C) with a heat map overlay of seed costs on the left. On the right is the Pareto front highlighting the mixtures where biomass and evenness are optimized.

We simulated the biomass production of 10,418 different polycultures using Eq. 5 and plotted their total crop biomass and species evenness (Fig. 6). The Pareto front was used to identify the seeding rate and species combinations that optimized these two parameters. On it, a tradeoff between evenness and biomass was observed, in which mixtures that had the highest levels of evenness did not produce the most biomass. No four‐species polycultures were found on the Pareto front and all polyculture combinations contained pearl millet (MSH, MSC, and MHC). The MSH polyculture was the most common with combinations that ranged from producing 398 g m^−2^ of biomass with an evenness of 1 to combinations producing 498 g m^−2^ of biomass with an evenness of 0.39.

Seeding rates and seed costs were examined for polycultures with an evenness of 0.8 or greater (Table 3). The MSH polyculture combinations that fit these criteria contained pearl millet proportions between 41 and 64% of its monoculture seeding rate, sorghum sudangrass proportions between 12 and 36% of its monoculture seeding rate, and sunn hemp proportions between 22 and 60% of its monoculture seeding rate. On average, these polycultures produced 436 g m^−2^ of biomass with an evenness of 0.92 at a cost of $279.40 ha^−1^. Based on these results, some general seeding rate principles emerged. Because sorghum sudangrass is much more competitive than pearl millet, sorghum sudangrass density should be lower than pearl millet to achieve high evenness. Because sunn hemp seed is relatively expensive, sunn hemp should be included at a low proportion if low seed costs are a priority.

**Table 3:** Polycultures identified on the Pareto front that had a species evenness greater than 0.8 ordered by decreasing total biomass and increasing evenness.

Mixture	Proportion of monoculture rate (%)				Total proportions	Biomass (g m^−2^)				Total biomass	Evenness	Cost
	M	S	H	C		M	S	H	C	(g m^−2^)		(US$ ha^−1)^
MSH	0.47	0.36	0.33	—	1.15	104.75	296.05	61.22	—	462.01	0.81	270.95
MSH	0.64	0.30	0.22	—	1.16	145.56	271.96	42.16	—	459.68	0.81	224.72
MSH	0.41	0.36	0.38	—	1.15	93.33	293.34	71.88	—	458.55	0.82	288.00
MSH	0.58	0.30	0.27	—	1.15	134.77	269.22	53.05	—	457.04	0.84	241.77
MSH	0.52	0.30	0.33	—	1.15	123.58	266.53	64.09	—	454.20	0.86	258.82
MSH	0.47	0.30	0.38	—	1.15	111.96	263.90	75.28	—	451.13	0.87	275.87
MSH	0.41	0.30	0.49	—	1.20	97.98	255.91	95.36	—	449.25	0.89	314.76
MSH	0.58	0.24	0.33	—	1.15	144.34	231.85	67.25	—	443.45	0.90	246.68
MSH	0.52	0.24	0.38	—	1.15	132.53	229.37	79.02	—	440.91	0.92	263.73
MSH	0.47	0.24	0.49	—	1.20	117.83	221.84	100.02	—	439.69	0.94	302.62
MSH	0.41	0.24	0.55	—	1.19	105.22	219.56	111.92	—	436.70	0.94	319.67
MSH	0.58	0.18	0.38	—	1.15	155.37	188.30	83.14	—	426.82	0.95	251.60
MSH	0.52	0.18	0.49	—	1.20	139.86	181.56	105.16	—	426.58	0.98	290.49
MSH	0.47	0.18	0.55	—	1.19	127.03	179.52	117.71	—	424.27	0.98	307.54
MSH	0.58	0.12	0.49	—	1.20	164.47	133.19	110.86	—	408.52	0.99	278.36
MSH	0.52	0.12	0.55	—	1.19	151.44	131.55	124.14	—	407.13	1.00	295.41
MSH	0.47	0.12	0.60	—	1.19	137.79	129.95	137.63	—	405.37	1.00	312.46
MSH	0.41	0.12	0.55	—	1.08	129.70	135.42	132.87	—	397.99	1.00	285.82
MHC	0.12	—	0.22	0.56	0.89	87.03	—	90.28	89.11	266.43	1.00	316.77
MHC	0.06	—	0.11	0.22	0.39	60.42	—	61.16	61.81	183.40	1.00	136.40

Notes

M, pearl millet; S, sorghum sudangrass; H, sunn hemp; C, cowpea.

## DISCUSSION

### Competitive traits of cover crop species

Sorghum sudangrass dominated the other species in mixtures in terms of biomass production. Similar asymmetrical competition by sorghum sudangrass was seen in the model validation experiment (Bybee‐Finley et al. 2016). As conditions were generally not nutrient limited, especially not N limited, or water scarce, competition was most probably driven by light availability.

Taller‐statured species were better able to capture light, sometimes instigating other species, such as pearl millet, to grow larger than they would have in monoculture or when paired with smaller‐statured species. Terao et al. (1997) described how the spreading growth habit of cowpea allowed for more light capture, particularly red:far red light, when being shaded by a more competitive plant. Cowpea leaves were generally larger but fewer in number than the sunn hemp leaves that grew from a main stem that was often taller than the cowpea, allowing it to capture more light. The heavier, rockier soil conditions probably limited the overall growth of cowpea and the rate of fertilizer probably had a negative impact on the RC ability of the tap‐rooted legumes (Gregory and Reddy 1982, Sheahan 2012).

The ability to better capture additional resources can be proxied using the crop growth rate. In the validation experiment, grasses had faster growth rates than either legume, and sunn hemp had faster growth rates than cowpea in both monocultures and mixtures (Bybee‐Finley et al. 2016). In addition to the faster growth rate, sorghum sudangrass has known allelopathic properties that suppress the growth of other species. Living root hairs continually excrete sorgoleone into the rhizosphere, although this has been shown to decrease as the plant matures (Weston and Czarnota 2008, Weston and Alsaadawi 2013). Larger plant size and faster growth rate, in addition to allelopathic traits probably gave the sorghum sudangrass a competitive advantage compared with the other four species.

Theoretical maximum weights (1/*b*_1,0_) can be used to discern a species potential in a mixture, but should be considered with seeding rates and competition coefficients. For example, cowpea had a large theoretical maximum, but this did not result in high biomass because of the low seeding density (recommended seeding rates of C were approximately 1/4, 1/3, and 1/2 of M, S, and H rates, respectively) and high degrees of intraspecific and interspecific competition. Pearl millet and sunn hemp both had greater theoretical weights when planted in bicultures with sorghum sudangrass than they did in monocultures, showing how interspecific competition can instigate the growth of the less competitive species. Pearl millet and sunn hemp had similar or lower intraspecific competition than sorghum sudangrass in bicultures yet increasing the seeding rate of sorghum sudangrass led to greater increases in biomass because of the larger size of sorghum sudangrass. Despite sunn hemp being a theoretically smaller plant when planted with pearl millet, increasing the seeding rate of sunn hemp led to a greater increase of biomass than increasing the seeding rate of pearl millet as pearl millet faced greater intraspecific competition than sunn hemp.

Generally, biomass in this experiment was similar to previous experiments in upstate New York (Brainard et al. 2011, Bybee‐Finley et al. 2016), but greater biomass production was seen in warmer locations and when species were planted at higher seeding rates (Creamer and Baldwin 2000, Balkcom and Reeves 2005, Schomberg et al. 2007, Finney et al. 2009, Blanco‐Canqui et al. 2011, Blanco‐Canqui and Claassen 2012). Differences in biomass production and competitive dynamics in mixtures are affected by the genetics of the species, environment, and management interactions (G × E × M). Growing conditions are likely to have affected the competitive traits of the four species. For example, a warmer, low‐N environment with sandier soil would probably be a more favorable environment for legumes than the conditions found in this experiment (Louarn et al. 2020). Multienvironment agronomic experiments such as those undertaken by Mirsky et al. (2017) that examined hairy vetch planting dates and seeding rates along the east coast and Schomberg et al. (2007) that examined planting and harvest dates of sunn hemp in the southeastern United States would be necessary to understand how environmental conditions and management decisions are related to competition. Future work should explore competition in cover crop mixtures across varying environments to assess the robustness of competition coefficients.

### Complementarity

We did not find support for our first hypothesis that functionally diverse bicultures would produce greater biomass and have a higher NDI than functionally similar bicultures. The driving mechanism of complementarity in annual crop mixtures is thought to be resource partitioning and is derived from the species traits that enable them to access resources in some unique way (Brooker, 2015, Bybee‐Finley and Ryan 2018). Resource partitioning is attributed to the functional diversity of species fitting into particular niches (Finney and Kaye, 2017). However, we were unable to relate functional type of the species to competition, although our experiment tested a limited range of functional types. Perhaps because characteristics of cover crop species have been selected for crop production, and mixtures have a shared, single season of growth, species are likely to have accessed available resources in a fairly similar manner. The traits driving light competition (e.g. light interception) may be more important to determine complementarity in annual mixtures in temperate agricultural fields with adequate rainfall and fertility. Future work could explore the physiological mechanisms of competition in crop mixtures, such as experiments that could measure light competition using crop growth rates, heights, or leaf area index (LAI), and root traits using rooting depth and root length density.

More biomass was produced in mixtures in which species faced lower interspecific competition than intraspecific competition. The biculture treatments with the greatest NDI values all contained pearl millet, suggesting that pearl millet is an ideal species to mix with the other species that were studied. Possible reasons for this include: (1) a smaller stature than sorghum sudangrass that reduced the amount of light competition for the other species in the biculture, (2) a faster crop growth rate (Bybee‐Finley et al. 2016) that allowed it to keep pace with sorghum sudangrass, (3) its fibrous root system, and (4) its plasticity in shoot and root development. Gregory and Reddy (1982) described pearl millet's root growth pattern as multiaxial with axes that grew at varying angles from the plant allowing for a considerable degree of mixing root systems when intercropped. Roots of pearl millet have also been shown to have the ability to grow to various lengths depending on growing conditions and intercropped species (Gregory 1986, Kizito, 2006). Although it had a relatively higher sowing density compared with other species, density appeared to be less of a factor for biomass production for pearl millet, perhaps because of its quick ability to adapt to growing conditions by vigorous tillering (Azam‐Ali and Gregory 1984). Barot et al. (2017) in a review of ecological mechanisms for crop mixtures suggested that plasticity in belowground and aboveground growth pre‐empts greater resource capture. Further research is needed to verify the physiological characteristics that resulted in the complementary nature of pearl millet.

### Predictive ability of competitive traits for higher order mixture

We found support for our second hypothesis that we can apply the competition coefficients estimated in the bicultures to accurately predict the biomass of three‐species and four‐species polycultures. This finding means that experiments on cover crop mixtures would only need to examine combinations of two species at various rates to understand outcomes for mixtures containing three or four species. Using Eq. 5 parameterized from our restricted response surfaces with bicultures, we were able to estimate seeding rates for species grown in polycultures that produced high biomass distributed evenly across species at low costs.

It is unclear whether this framework, particularly Eq. 5, would be as accurate in higher diversity mixtures. While we were able to show that Eq. 5 worked well with predicting biomass from four‐species polycultures across a range of environments, it is likely that higher diversity mixtures would necessitate the replication of the response surface to ensure the accuracy of the competition coefficients. Competition coefficients could be made more robust if developed under different growing conditions. By understanding how key aspects of a G × E × M gradient (e.g. a soil type or a latitude) interacted with the model, the amount of deductive research required to design cover crop mixtures for various conditions could be further reduced.

Our framework is premised on functional diversity (i.e., delivery of ecosystem services by species with different functions) and not response diversity (i.e., reducing the risk of losing ecosystem services by having varied responses to growing conditions) (Elmqvist et al. 2003). Because the framework measures the responses of the species, these two categories of diversity are inseparable. Yet, practitioners are often motivated to plant multiple species in precaution to unknown conditions. This framework does not consider the potential benefits that functional redundancy provides (Leslie and McCabe 2013). Additional research should examine multiple levels of redundancy within functional groups to improve the understanding of the effects of response diversity.

### Designing mixtures for multifunctionality

Previous work has suggested that to reduce such asymmetric competition in cover crop mixtures, the seeding rates of highly competitive species should be reduced and the seeding rates of less competitive species should remain near their monoculture rates (White, 2015). However, our framework is the first to describe this process based on empirical models. The MSH polycultures had the greatest multifunctionality based on our criteria. In the validation experiment, the MSH polyculture was also the most even treatment at 45 DAP. It also maintained or increased evenness at 90 DAP, suggesting that more symmetric competition earlier in the growing season results in more evenness later (Bybee‐Finley et al. 2016). It is important to realize that thresholds for species evenness have not been well established and highlight a knowledge gap.

The total proportion of the seeding rates for the MSH polycultures with evenness > 0.8 was more than 100%, indicating that, to achieve multiple objectives, seeding rates greater than replacement rates may be necessary. Although, the higher seeding rates identified on the Pareto front are partially an artifact of the upper bound that is set on the optimization data frame to be within our experimental densities and the asymptotic property of Eq. 5 (i.e., an increasing density will continue to produce an increasing amount of biomass). The seeding densities used for the bicultures were mostly lower than the recommended monoculture seeding rates (cumulative proportions ≤ 1) and we assumed that the yield–density relationship remained asymptotic. To determine the density at which the yield–density relationship plateaus, seeding rates in experimental treatments would need to increase well beyond the recommended monoculture seeding rates (Kikuzawa 1999, Li and Hara 1999).

## Conclusion

Cover crops are the main crop diversification practice supported by agriculture policies in the United States because their implementation requires minimal changes to current cropping systems compared with other diversification practices, such as expanded crop rotations. Yet, adoption of cover crops remains limited, due in part to high seed costs (Wayman et al. 2017, Bergtold et al. 2019). The goal of cover crop mixtures is often to deliver multiple ecosystem services beyond what can be provided by a single species (Isbell, 2011). These additional ecosystem services consequently provide stability and resilience to the agro‐ecosystem (Loreau and de Mazancourt 2013). As seeding rates are an integral factor for biomass production and competition, precisely constructed cover crop mixtures can enhance the delivered ecosystem services in a cost‐effective manner. Our framework describes the process of determining the seeding rates of species to achieve greater multifunctionality in the agro‐ecosystem. Decision‐making tools, such as cover crop calculators,^6^ could integrate this framework into their design and provide practitioners with an improved ability to use cover crops to provide multiple ecosystem benefits and achieve more sustainable cropping systems.

## Supporting information

Appendix S1

## Data Availability

Data and code are permanently archived on Cornell University digital repository eCommons: https://doi.org/10.7298/bvbq‐6z48

## Footnotes

^6^

https://smartmix.greencoverseed.com.
