# Supplementary material for: Finding the right mix: a framework for selecting seeding rates for cover crop mixtures
Source: Ecol Appl. 2021 Nov 24;32(1):e02484. doi: 10.1002/eap.2484 (PMC9285019; doi:10.1002/eap.2484)
Supplement: Supplementary file 1 — Appendix S1 [file EAP-32-0-s001.pdf]

Supporting Information. Bybee-Finley, K.A., S. Cordeau, S. Yvoz, S.B. Mirsky, and M.R. Ryan. 2021. Finding the right mix: a framework for selecting seeding rates for cover crop mixtures. Ecological Applications.

Appendix S1

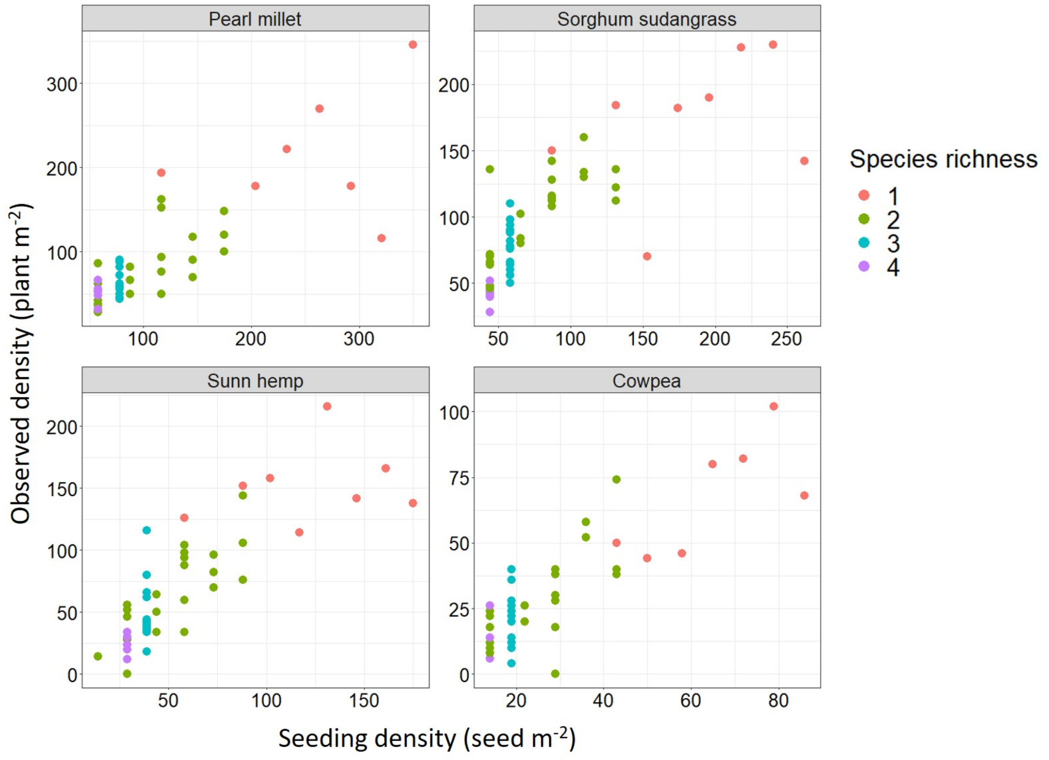

Figure S1. The intended seeding density of each species by the observed density. Monoculture seeding rates of a species were typically greater than those of mixtures. The density of cowpea and sunn hemp generally increased with higher seeding rates. However, the density of the two grasses, particularly pearl millet, responded with greater variability to increased seeding rates.

$$\frac{1}{W_1} = \frac{N_1}{Y_{1,2}} = b_{1,0} + b_{1,1} * N_1 + b_{1,2} * N_2 \qquad \text{(Eq. S1)}$$

The linear relationship of the yield-density model of a two-species mixture (Eq 2), in which W_1 is the per plant weight of species *1* in a biculture of species *1* and *2*. N_1 and N_2 are the plant densities of each species and $Y_{1,2}$ is the biomass of species *1* in the biculture. The term $b_{1,0}$ is the intercept. The term $b_{1,1}$ (m^2 g^{-1}) is a measure of intraspecific competition and describes how the per plant weight (W_1) decreases when additional plants of the same species are added. The term $b_{1,2}$ (m^2 g^{-1}) is a measure of interspecific competition of species *2* on species *1* and describes how the per plant weight (W_1) decreases when plants of species *2* are added.

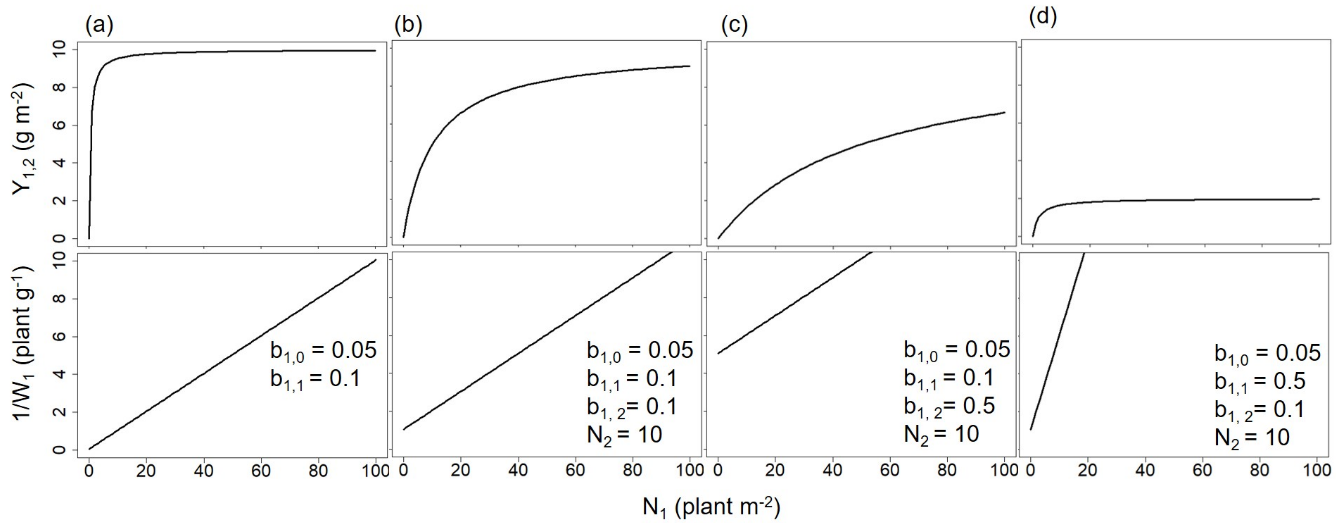

Figure S2. Different competition scenarios of the yield-density relationship between species, *1* and *2*, as described by Eq 2. The upper panels represent the hyperbolic relationship between biomass and density and the lower panels represent the linear reciprocal of the hyperbolic model. a) As species *2* is absent, only intraspecific competition is present. b) Intra- and interspecific competition are the same. c) Intraspecific competition remains the same as in (a) and (b), while interspecific competition has increased; as a result the slope of the line is the same as in (a) and (b), but the *y* intercept of the linear model has shifted upwards. d) Intraspecific competition has increased, while interspecific competition is the same as in (b); as a result, the slope of the linear model has increased, but the *y* intercept is the same as in (b). $Y_{1,2}$ is the biomass (g m^{-2}) of species *1* in a biculture with species *2*, N is the density (plant m^{-2}), $b_{1,0}$ is the intercept, $b_{1,1}$ represents intraspecific competition, $b_{1,2}$ represents the interspecific competition. To focus on the changes from competition, $b_{1,0}$ was kept constant in all scenarios, although this is unlikely to occur.

Table S1. Raw competition coefficients and their standard errors, as determined by Eq 1 and Eq 2.

Treatments	Species	b1,0 (plant g^{-1})	SE	b1,1 (m^2 g^{-1})	SE	b1,2 (m^2 g^{-1})	SE
M	M	0.1336	0.1290	0.0028	0.0006		
S	S	0.0491	0.0160	0.0019	0.0001		
H	H	0.1606	0.0728	0.0021	0.0005		
C	C	0.0930	0.0166	0.0033	0.0166		
MS	M	0.1319	0.0950	0.0028	0.0005	0.0082	0.0023
MS	S	0.0658	0.0241	0.0018	0.0002	0.0002	0.0002
MH	M	0.1646	0.0922	0.0027	0.0005	0.0029	0.0012
MH	H	0.2091	0.0577	0.0018	0.0004	0.0013	0.0004
MC	M	0.1920	0.0902	0.0026	0.0005	0.0008	0.0022
MC	C	0.0970	0.0145	0.0033	0.0002	0.0033	0.0004
SH	S	0.0839	0.0267	0.0017	0.0002	0.0007	0.0003
SH	H	0.1502	0.0548	0.0022	0.0004	0.0043	0.0007
SC	S	0.0644	0.0260	0.0018	0.0002	0.0015	0.0006
SC	C	0.1007	0.0218	0.0032	0.0004	0.0041	0.0007
HC	H	0.1548	0.0430	0.0022	0.0003	0.0017	0.0010
HC	C	0.0931	0.0128	0.0033	0.0002	0.0034	0.0004

The model for the bicultures generated intraspecific competition coefficients that were quite similar to the intraspecific coefficients generated by the monocultures. Thus the decrease of per plant weight from an additional plant of the same species in a biculture was similar to the rate in monoculture. In some instances, some of the ranges of intraspecific competition (coefficient +/- SE) of the bicultures did fall outside of the monoculture ranges (Appendix S1: Table S1). The pearl millet in the MC biculture had a lower competition coefficient than in monoculture, meaning an additional pearl millet plant in biculture did not cause as much of a decrease in per plant weight as an additional plant in monoculture. The same is true for sunn hemp in the MH biculture and cowpea in the SC biculture. Cowpea in the MC biculture had a greater range of intraspecific competition coefficients, signaling the potential for an increased or decreased rate in

per plant weight with each additional plant as compared to cowpea in monoculture. Similarly, sorghum sudangrass had a greater range of intraspecific competition in the MS and SC bicultures than in monoculture but a reduced decrease in per plant weight in the SH biculture.

Table S2. The metrics for assessing model fits for Figure 5 of each species in the nine site-years, which took place in three locations over two years, in which some sites had experiment replicates. M, pearl millet; S, sorghum sudangrass; C, cowpea; H, sunn hemp; RMSE, root mean square error; slope, the coefficient of correlation between observed and predicted; *P* value (slope ≠1) describes if the model predictions had bias; r^2, the coefficient of determination describes the strength of the correlation.

Site	Species	No. of observations	RMSE	Slope	*P* value (slope≠1)	r^2
AurL13	M	17	1.10	0.36	0.02	0.13
	S	18	1.56	-0.29	<0.001	0.08
	H	18	1.09	0.37	0.02	0.14
	C	17	1.41	-0.06	<0.001	0.003
AurE14	M	16	0.53	0.85	0.30	0.72
	S	16	0.76	0.69	0.13	0.48
	H	16	0.66	0.76	0.19	0.58
	C	16	0.50	0.87	0.34	0.76
AurL14	M	15	0.73	0.71	0.16	0.51
	S	15	0.95	0.52	0.06	0.27
	H	16	0.91	0.56	0.07	0.31
	C	15	0.76	0.69	0.15	0.48
BelE13	M	16	0.71	0.73	0.16	0.54
	S	16	0.66	0.77	0.20	0.59
	H	16	0.91	0.56	0.07	0.32
	C	16	0.69	0.75	0.18	0.56
BelL13	M	16	0.76	0.69	0.13	0.48
	S	16	0.77	0.68	0.13	0.47
	H	16	0.58	0.82	0.26	0.68
	C	16	0.48	0.88	0.36	0.77
BelL14	M	16	0.58	0.82	0.26	0.67
	S	16	0.98	0.49	0.05	0.24
	H	16	0.67	0.76	0.19	0.58
	C	16	1.15	0.30	0.02	0.09
WilL13	M	14	0.58	0.82	0.29	0.67
	S	14	0.63	0.79	0.26	0.62
	H	15	0.71	0.73	0.18	0.53
	C	15	1.04	0.42	0.04	0.17

WilE14	M	24	1.21	0.23	0.001	0.05
	S	24	1.06	0.42	0.01	0.17
	H	24	0.75	0.71	0.07	0.50
	C	24	0.75	0.71	0.07	0.50
WilL14	M	24	0.62	0.80	0.13	0.64
	S	24	0.70	0.74	0.08	0.55
	H	24	0.91	0.57	0.02	0.32
	C	24	0.97	0.51	0.01	0.26
